# Exploiting the WH/ZH symmetry in the search for new physics

**DOI:** 10.1140/epjc/s10052-018-6234-x

**Published:** 2018-09-21

**Authors:** R. V. Harlander, J. Klappert, C. Pandini, A. Papaefstathiou

**Affiliations:** 1 TTK, RWTH Aachen University, 52056 Aachen, Germany; 2 EP Department, CERN, 1211 Geneva 23, Switzerland; 3 Institute for Theoretical Physics Amsterdam and Delta Institute for Theoretical Physics, University of Amsterdam, Science Park 904, 1098 XH Amsterdam, The Netherlands; 4 Nikhef, Theory Group, Science Park 105, 1098 XG Amsterdam, The Netherlands

## Abstract

We suggest to isolate the loop-induced gluon-initiated component ($gg\rightarrow ZH$) for associated $ZH$ production by using the similarity of the Drell–Yan-like component for $ZH$ production to the $WH$ process. We argue that the cross-section ratio of the latter two processes can be predicted with high theoretical accuracy. Comparing it to the experimental $ZH/WH$ cross-section ratio should allow to probe for new physics in the $gg\rightarrow ZH$ component at the HL-LHC. We consider typical BSM scenarios in order to exemplify the effect they would have on the proposed observable.

## Introduction

The Higgs boson provides a new probe for physics beyond the standard model (SM). A precise measurement of its couplings to the SM particles is certainly one of the most promising ways to search for deviations from the SM. The tree-level couplings of the SM Higgs are determined solely by the particle masses and the vacuum expectation value $v\approx 246$ GeV; a global fit to these couplings yields good agreement with the SM predictions within current experimental uncertainties, see e.g. Ref. [1]. Couplings to massless particles like the photon or gluons are necessarily loop-induced, which allows for new physics to affect the numerical value or the Lorentz structure of these couplings in a significant way.

In fact, the loop-induced couplings to photons as well as to gluons were essential to the actual discovery of the Higgs boson, for example through $gg\rightarrow H\rightarrow \gamma \gamma$ [2, 3]. The good agreement with the theoretical prediction of this process within the SM leaves little room for any large impact of new physics here (for comprehensive reviews on Higgs physics at the Large Hadron Collider (LHC), see Refs. [4–7]).

Associated $VH$ production, or Higgs-Strahlung for short, is one of the main production modes for Higgs bosons at the LHC. Despite its rather small cross section, its feature of providing a tag through the electro-weak gauge boson in the final state recently allowed the first observation of the Higgs decay to bottom quarks, which is swamped by background $b\bar{b}$ production in other major Higgs production modes. Focusing on boosted-Higgs events and advanced jet-substructure analyses is a promising direction to further separate the signal from the background [8].

**Fig. 1:**
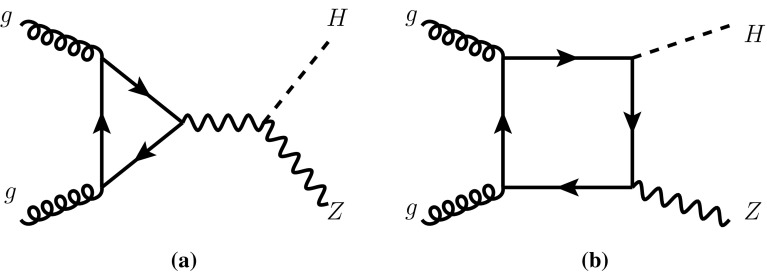
Sample Feynman diagrams that contribute to the $gg\rightarrow ZH$ process at leading order

Another unique feature of the Higgs-Strahlung process $pp\rightarrow VH$ is its appearance in two variants: $V=W$ and $V=Z$. In the SM, the amplitudes are related through next-to-leading order (NLO) QCD by well established symmetry properties of the SM. At next-to-next-to-leading order (NNLO) QCD, however, these two concepts are no longer sufficient to relate $WH$ to $ZH$ production. This is mostly due to a loop-induced contribution to $ZH$ production whose leading-order (LO) partonic amplitude is given by $gg\rightarrow ZH$. The corresponding Feynman diagrams contain either boxes or triangles of bottom or top quarks, see Fig. 1 (lighter-quark contributions are numerically negligible in general). In the SM, the box and triangle contributions interfere destructively, which leads to an enhanced sensitivity to physics beyond the SM (BSM).

Quite generally, loop-induced processes are particularly sensitive to new physics, since its effects are likely to be of the same order as the SM process in this case. The sub-process $gg\rightarrow ZH$, however, is only one contribution to the general Higgs-Strahlung process of $ZH$ production, albeit a separately-finite and gauge-independent one. Moreover, it is suppressed by two powers of the strong coupling constant $\alpha _s$ with respect to the dominant $q\bar{q}$-initiated contribution.

It would thus be desirable to separate event samples which are due to the “Drell-Yan-like” production mechanism, where at LO the Higgs is radiated off an off-shell *Z* boson produced in $q\bar{q}$ annihilation, from the ones due to the gluon-initiated process $gg\rightarrow ZH$. First steps in this direction have been taken in Refs. [9, 10]. In the latter paper, it was pointed out that the relative contribution of the $gg\rightarrow ZH$ process to $ZH$ production depends strongly on the kinematical region of the final state. For example, while it constitutes only about 6% of the total cross section (at order $\alpha _s^2$), its relative contribution is more than twice as large in the so-called boosted regime, $p_T>150$ GeV. Clearly, such an effect needs to be taken into account in experimental analyses of the $ZH$ production process, in particular since it carries such a large sensitivity to new physics.

In this paper, we propose a data-driven strategy to extract the gluon-initiated component (or, more precisely, the non-DY component) for $ZH$ production. It is based on the comparison of the $ZH$ to the $WH$ cross section and the corresponding invariant mass distribution of the $VH$ system. The required theory input in the SM is the ratio of the DY-like components for $ZH$ and $WH$ production, which can be predicted very reliably already now, and is expected to improve even further in the foreseeable future. We study the impact of various possible structures in models for new physics, such as modified Yukawa couplings, extended Higgs sectors, or vector-like quarks (VLQs). In order to estimate the expected experimental uncertainties, we simulate a recent ATLAS analysis with Monte-Carlo events, and extrapolate it to higher luminosities. We find that the estimate of systematic uncertainties becomes the limiting factor for the measurement, highlighting the importance of a detailed investigation of systematic effects, and potentially an optimization of the experimental analysis towards the extraction of this ratio from data.

## Theory prediction for *VH* production

### Definition and features

Let us consider the following theoretical decomposition of the inclusive $VH$ production cross section:

1 $$\begin{aligned} \begin{aligned} \sigma ^{VH} = \sigma ^{VH}_{\text {DY}} + \sigma ^{VH}_\text {non-DY}, \end{aligned} \end{aligned}$$

where, by definition, the DY component can be written as

2 $$\begin{aligned} \begin{aligned} \sigma ^{VH}_{\text {DY}} = \int \mathrm{d}q^2\,\sigma _{V}(q^2)\,\frac{\mathrm{d}\Gamma _{V^*\rightarrow VH}}{\mathrm{d}q^2} + \Delta \sigma ^{VH}_\text {EW}. \end{aligned} \end{aligned}$$

In Eqs. (1) and (2), the electro-weak corrections $\Delta \sigma ^{VH}_\text {EW}$ are understood to be fully attributed to $\sigma ^{VH}_{\text {DY}}$, i.e., by definition, $\sigma ^{VH}_\text {non-DY}$ does not receive any electro-weak corrections. At LO perturbation theory, the DY-like terms for $WH$ are related to those for $ZH$ by changing external parameters like the gauge boson mass, the gauge coupling, or the PDF, all of which can (and are) determined independently through other processes. The effect of higher orders on this similarity between the DY components will be studied below. Note that any New Physics most likely respects the well-established gauge symmetry between the *W* and the *Z*, and thus preserves the strong tie between the DY-components for $WH$ and $ZH$ production. For example, in a general 2-Higgs-Doublet-Model (2HDM), the ratio of the DY components for $ZH$ and $WH$ production is the same as in the SM.

Concerning $\sigma ^{VH}_\text {non-DY}$, the dominant contribution in the SM for $V=Z$ is due to the gluon-initiated process $gg\rightarrow ZH$, denoted by $\sigma _{gg}$. The generic set of diagrams contributing to this sub-process at LO is shown in Fig. 1. We stress that, within QCD, $\sigma _{gg}$ is well-defined since it is separately finite and gauge invariant to all orders of perturbation theory. In BSM theories, also $b\bar{b}$-initiated contributions may become important in $\sigma ^{ZH}_\text {non-DY}$. None of these have a correspondence in $WH$ production; in fact, in this paper we will assume that only $ZH$ production receives non-DY contributions, i.e. $\sigma ^{WH}_\text {non-DY}=0$.

The current theoretical precision is quite different for the first two components on the l.h.s. of Eq. (1). While $\sigma ^{VH}_{\text {DY}}$ is known through NNLO QCD [11–15], i.e. ${\mathcal O}(\alpha _s^2)$, the current theory prediction for the total inclusive cross section of $\sigma _{gg}$ is based on the full LO calculation, which is also of order $\alpha _s^2$ [16, 17]. At this order, $\sigma _{gg}$ amounts to about 6% of the total $ZH$ cross section for $M_\text {H}=125$ GeV in *pp* collisions at 13 TeV. A full calculation of the relevant NLO corrections, i.e. ${\mathcal O}(\alpha _s^3)$, is not yet available. However, assuming that it depends only weakly on the top-quark mass, as it is the case for the gluon-fusion process $gg\rightarrow H$, the NLO correction factor has been found to be of the order of two, which increases the $gg\rightarrow ZH$ contribution to the total cross section accordingly [18, 19]. Higher order terms in $1/M_\text {t}$ were evaluated in Ref. [20], but their validity is restricted to an invariant mass $M_{ZH}$ of the $ZH$ system of $M_{ZH}<2M_\text {t}$. Concerning differential distributions, the amplitudes for 2- and 3-parton final states including the full quark-mass dependence have been merged in order to obtain a reliable prediction at large transverse momenta of the Higgs boson [21, 22]. For $\sigma _{\text {DY}}^{VH}$, also electro-weak corrections are known [23–25], while they are unavailable for $\sigma _{gg}$ at the time of this writing. As a consequence, the estimated theoretical accuracy due to scale variation for the DY-like component is at the sub-percent level, while it reaches up to about 25% for $\sigma _{gg}$ at NLO. Including NLL resummation, this reduces to about 7% [19]. The PDF uncertainties^1^ are at the 2% and 4% level for the DY and the *gg* component, respectively. NNLO+PS implementations of the $WH$ and the $ZH$ process have been presented in Refs. [27, 28].

### New-physics effects

The gluon-initiated component reveals some interesting features which predestines it as a probe for new physics. First of all, it is loop-induced, which means that it is particularly sensitive to as-of-yet unknown particles which might couple the initial-state gluons to the $ZH$ final state. Second, the dominant contribution in the SM is due to top-quark loops, which lead to a characteristic threshold-structure in various kinematical distributions of the cross section. The application of appropriate cuts thus allows for enriching the $ZH$ sample with gluon-initiated events, as pointed out in Ref. [10]. Through the box diagrams, Fig. 1b, the cross section also receives a dependence on the top-quark Yukawa coupling, which is amplified by the fact that the box diagrams interfere destructively with the triangle diagrams, Fig. 1b. Another interesting feature which appears in many BSM models are *s*-channel contributions due to additional Higgs bosons [9]. They either add to the triangle-component of $\sigma _{gg}$, or they occur in the process $b{\bar{b}}\rightarrow ZH$. For future reference, we refer to the latter contribution as $\sigma _{b{\bar{b}}}$, distinguishing it from the $b{\bar{b}}$-contribution to $\sigma _{\text {DY}}^{VH}$ by requiring that $\sigma _{b{\bar{b}}}= 0$ in the limit of a vanishing bottom-quark Yukawa coupling. In the SM, this contribution amounts to less than 0.1% of the DY term.

Many of such New-Physics effects on $\sigma _{gg}$ as well as $\sigma _{b{\bar{b}}}$ can be investigated with the help of the program vh@nnlo [29, 30].

**Fig. 2:**
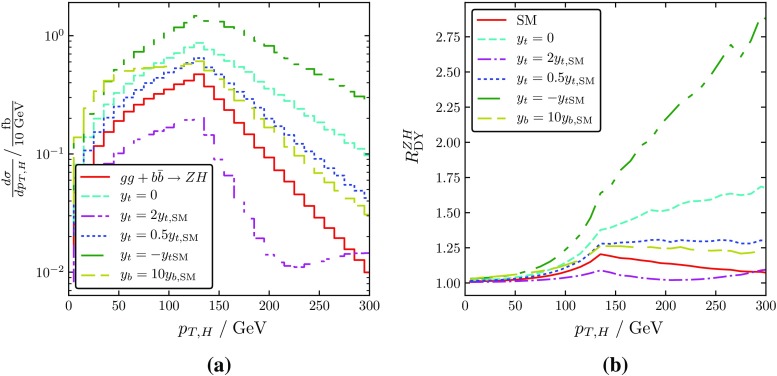
**a** The $p_T$ spectrum of the Higgs boson produced through the *gg*- and $b{\bar{b}}$-processes for different values of the top- and bottom-quark Yukawa couplings. **b** The ratio of the full $ZH$ cross section to the DY component. The latter was obtained at NNLO with the help of MCFM [15], the new-physics effects in $gg\rightarrow ZH$ were calculated at LO (i.e. ${\mathcal O}(\alpha _s^2)$) with vh@nnlo [29, 30], using PDF4LHC15_nnlo PDFs with $\alpha _s(M_Z)=0.118$ in both cases [26]. The (local) minimum at $p_T\sim 230$ GeV for $y_t=2y_{t,\text {SM}}$ is an effect from the box–triangle interference

Deviations from the SM, like modified Yukawa couplings, new colored particles, or an extended Higgs sector, are thus likely to manifest themselves in the $ZH$ final state through the gluon- or $b{\bar{b}}$-initiated component of the cross section, given that one considers a suitable observable. In Sect. 3 we will argue that the DY and the non-DY $ZH$ contribution can be isolated to a high degree by considering the ratio^2^

4 $$\begin{aligned} R^{ZH}_{\text {DY}} \equiv \frac{\sigma ^{ZH}}{\sigma ^{ZH}_{\text {DY}}}. \end{aligned}$$

An obvious kinematical parameter to consider would be the transverse momentum of the Higgs boson. Indeed, as shown in Fig. 2, the $p_T$ distribution of the Higgs boson produced in non-DY processes exhibits a significant dependence on new physics (a non-SM Yukawa coupling in this case).

**Fig. 3:**
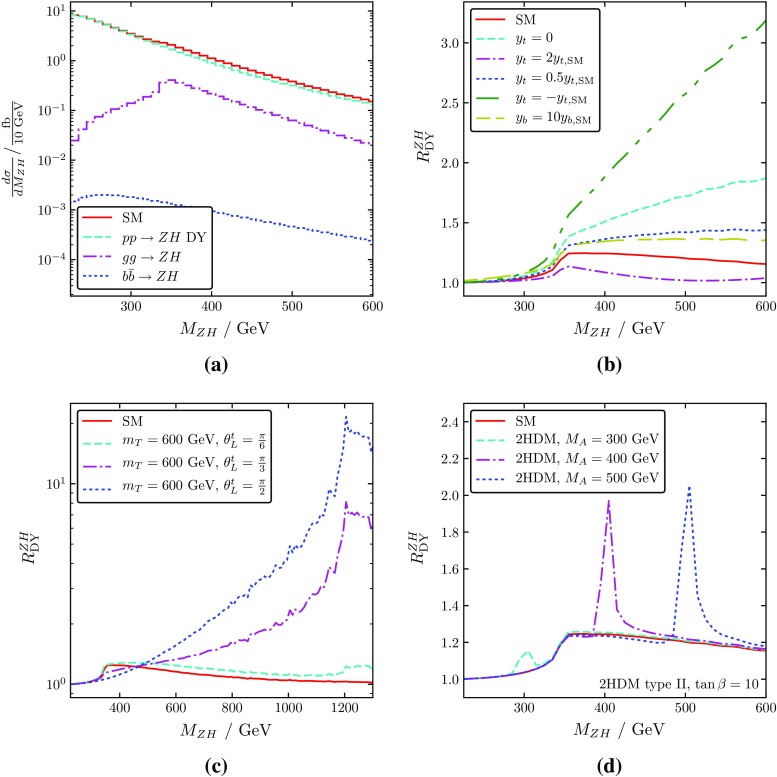
**a** The $ZH$ invariant mass spectrum in the SM: total cross section (solid red), DY component (dashed cyan), $gg\rightarrow ZH$ component (dash-dotted purple), $b{\bar{b}}\rightarrow ZH$ component (dotted blue). **b** The ratio $R_{\text {DY}}^{ZH}$ of the full $ZH$ cross section to the DY component in the SM (solid red) and for modified values of the top- and bottom-quark Yukawa coupling, including various new-physics effects. **c** $R_{\text {DY}}^{ZH}$ for the SM+VLQs of mass 600 GeV, and various values of the VLQ mixing angle. **d** $R_{\text {DY}}^{ZH}$ for the production of a light SM-like Higgs in the 2HDM with a pseudo-scalar of different masses including $b{\bar{b}}\rightarrow ZH$. The DY-like component was obtained at NNLO with the help of MCFM [15], the New-Physics effects in $gg\rightarrow ZH$ were calculated at LO (i.e. ${\mathcal O}(\alpha _s^2)$) with vh@nnlo [29, 30], using PDF4LHC15_nnlo PDFs with $\alpha _s(M_Z)=0.118$ in both cases [26]

In this paper, however, we want to focus on the invariant mass distribution $M_{ZH}$ of the $ZH$ system, since we find that it reveals particularly distinct features that allow to identify various New-Physics models, especially when normalized to the DY-like $ZH$ contribution. Examples are shown in Fig. 3, which include the effect of both $gg\rightarrow ZH$ and $b{\bar{b}}\rightarrow ZH$, the latter of which becomes relevant in scenarios with enhanced bottom-quark Yukawa coupling. Experimentally, the invariant mass for the $M_{VH}$ system may be difficult to access, and other observables such as the $p_T$ spectrum may be more advantageous. The optimal observable is best determined within an experimental analysis where all the systematic uncertainties are available. The reconstruction of the $WH$ invariant mass for our numerical simulation is outlined in Sect. 4. The general idea of the current paper is independent of the observable under consideration.

Throughout the paper, we set

5 $$\begin{aligned} \begin{aligned} \sqrt{s}=13\,\text {TeV},\quad M_\text {t}=173\,\text {GeV},\quad M_\text {H}=125\,\text {GeV}, \end{aligned} \end{aligned}$$

unless indicated otherwise. As already pointed out in Ref. [10], the contribution of $gg\rightarrow ZH$ to the total cross section is typically rather small in the kinematical region below the top-quark threshold. The distribution above $2M_\text {t}$, on the other hand, distinctly reflects the impact of New Physics. Specifically, this region crucially depends on the top-quark Yukawa coupling, both in magnitude and sign, as shown in Fig. 3a, b. In addition, new heavy particles which contribute to the effective *ggZH* coupling might also reveal extra threshold structures in the invariant mass spectrum, as shown using the example of vector-like quarks in Fig. 3c. Non-minimal Higgs bosons which contribute through *s*-channel exchange lead to yet other features in this spectrum, see Fig. 3d, which shows $R_{\mathrm {DY}}^{ZH}$ for a 2HDM. The peak structure is dominated by the $b{\bar{b}}\rightarrow ZH$ process in this case (see also Ref. [30]).

## Extracting the non-DY component from data

### The double ratio

The high accuracy to which the DY component is known theoretically suggests a simple comparison of the experimentally determined $VH$ rate to the theoretical prediction of its DY component, and thus the extraction of the non-DY to the DY ratio directly from $R_{\text {DY}}^{ZH}=\sigma ^{ZH}/\sigma ^{ZH}_{\text {DY}}$ of Eq. (4):

6 $$\begin{aligned} \begin{aligned} \frac{\sigma ^{ZH}_\text {non-DY}}{\sigma _\text {DY}^{ZH}} = R_{\text {DY}}^{ZH}-1 = \frac{\sigma ^{ZH}}{\sigma _{\text {DY}}^{ZH}} - 1, \end{aligned} \end{aligned}$$

with the DY-like cross section, $\sigma _\text {DY}^{ZH}$, taken from theory, and the full $ZH$ cross section $\sigma ^{ZH}$ from experiment. Such an experiment/theory comparison suffers from potential systematic uncertainties though, due to detector simulation, unfolding, and the like.

In this paper, we propose to analyze the data from Higgs–Strahlung by making use of a very specific feature for this process which has been alluded to in Sect. 2.1, namely the similarity between the $ZH$ and the $WH$ process.^3^ For this purpose, let us define the double ratio

7 $$\begin{aligned} \begin{aligned} R_R^{ZW} = \frac{\sigma ^{ZH}/\sigma ^{WH}}{\sigma ^{ZH}_{\text {DY}}/\sigma ^{WH}} \equiv \frac{R^{ZW}}{R_{\text {DY}}^{ZW}}. \end{aligned} \end{aligned}$$

Obviously, if all quantities are evaluated theoretically, it is $R_R^{ZW} = R^{ZH}_{\text {DY}}$, cf. Eq. (4). Here, however, we suggest to measure the numerator $R^{ZW}=\sigma ^{ZH}/\sigma ^{WH}$ of the double ratio in Eq. (7) from experimental data. Despite the different final states for $ZH$ and $WH$ production, we expect that a number of systematic experimental uncertainties cancel, in particular if the parameters of the analyses for $ZH$ and $WH$ are aligned as much as possible. A rough estimate of the experimental uncertainty will be described below.

The denominator of Eq. (7), on the other hand, referred to as the DY ratio in what follows, can be calculated within the SM with rather high precision, as will be discussed below. In addition, it can hardly be affected by any New-Physics effects, because of the strong theoretical and experimental constraints on the electro-weak gauge couplings, as already discussed in Sect. 2.1.

We note that the comparison of $WH$ to $ZH$ as a probe for New Physics has been first suggested in Ref. [9], where the 2HDM was considered as an example at the level of total cross sections, partly with boosted topology. In this paper we provide a much more elaborate investigation of that proposal, on the basis of differential quantities and including an estimate of the expected experimental uncertainty through the analysis of a simulated event sample.

### Theory prediction for the DY ratio

At the level of the total cross section, $R_{\text {DY}}^{ZW}$ receives corrections of only 0.2% at NLO, while the NNLO corrections on top of that are at the per-mill level. This is quite remarkable as the NLO corrections on the numerator and denominator in that ratio amount to 16%; the NNLO corrections on the other hand, are less than 1% on top of that.

**Fig. 4:**
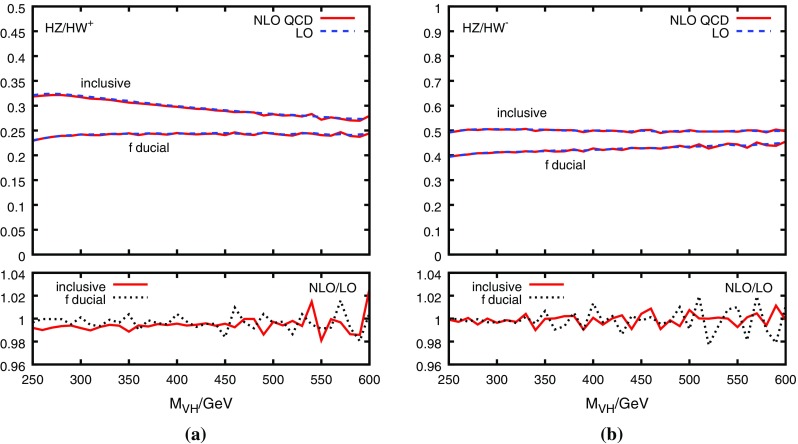
QCD corrections (including $qg\rightarrow qVH$) to the ratio $R^{ZW}_{\text {DY}}$ for **a** $W=W^+$ and **b** $W=W^-$ as a function of the $VH$ invariant mass, $V\in \{Z,W\}$. The dashed/solid line in the upper parts of the plots show the LO/NLO QCD result, the lower parts show the ratio of the two. Obtained with HAWK [24, 32] (only the decays $Z\rightarrow l^+l^-$ and $W\rightarrow l\nu$ are included) using NNPDF23_qed_nlo PDFs with $\alpha _s(M_Z)=0.118$ [39]

As a function of $M_{VH}$, the NLO corrections on the DY-ratio are at or below the 1% level, as shown in Fig. 4. This holds for both the fully inclusive as well as the “fiducial” cross section, where the latter is evaluated according to Ref. [7] by applying the following cuts:

8 $$\begin{aligned} \begin{aligned} p_T^\ell > 15\,\text {GeV},\quad y_\ell<2.5,\quad 75\,\text {GeV}< m_{\ell \ell } <105\,\text {GeV}, \end{aligned} \end{aligned}$$

where $p_T^\ell$ and $y_\ell$ is the transverse momentum and rapidity of a charged lepton, respectively, and $m_{\ell \ell }$ is the invariant mass of a charged lepton pair (the latter cut only applies to $ZH$ production, of course). Using MCFM, we have also checked that the NNLO corrections on the DY-ratio are negligible for all relevant values of $M_{VH}$. For the NLO prediction, we thus estimate the uncertainty due to uncalculated QCD corrections to be less than 1%.

**Fig. 5:**
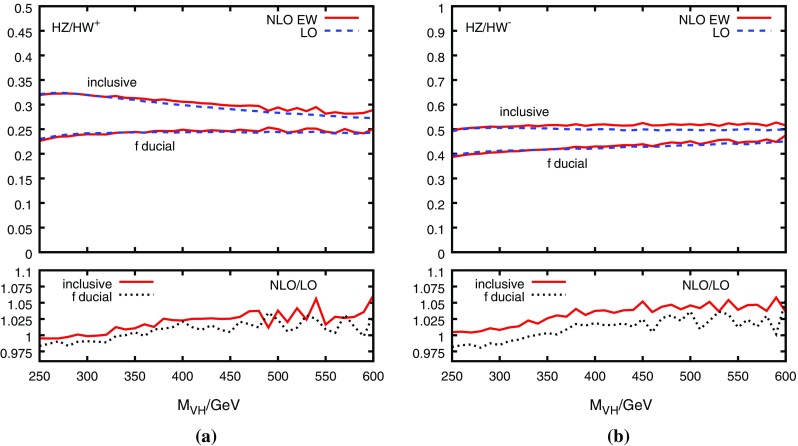
**a**, **b** Same as Fig. 4, but for electro-weak corrections (*ex*cluding $\gamma q\rightarrow qVH$)

Due to the different electric charge of *W* and *Z* and their different decay patterns, one may expect a larger sensitivity of the ratio $R_{\text {DY}}^{ZW}$ to electro-weak corrections in comparison to the QCD effects. Indeed, employing HAWK [24, 32] to study these effects, we find that they amount up to about 5% on $R_{\text {DY}}^{ZW}$ when the *Z* decay into charged leptons is considered,^4^ see Fig. 5. Compared to the QCD corrections, the electro-weak effects on $R_{\text {DY}}^{ZW}$ show a stronger dependence on $M_{VH}$, albeit in a very continuous and monotonous way.

**Fig. 6:**
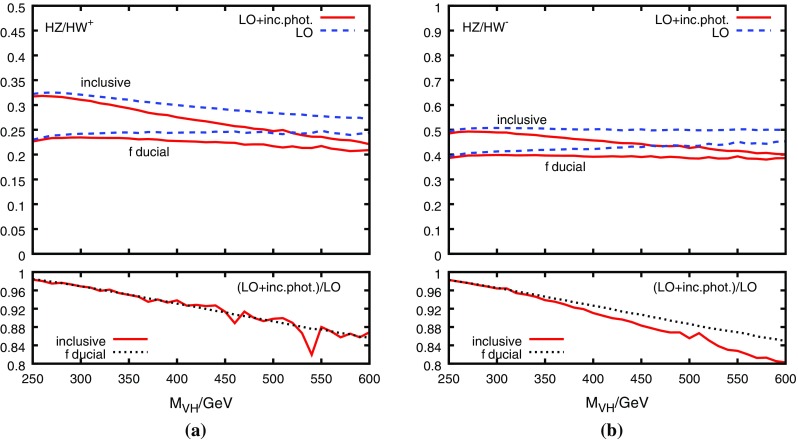
Same as Fig. 5, but for photon-induced corrections $\sigma _\gamma$, i.e. $\gamma q\rightarrow qVH$, and using LUXqed_plus_PDF4LHC15 PDFs with $\alpha _s(M_Z)=0.118$ [26, 33]

A particularly subtle electro-weak contribution is due to the photon-induced process $\gamma q\rightarrow qVH$, referred to as $\sigma _\gamma$ in what follows. Despite the fact that $\sigma _\gamma$ amounts to at most about 7% to the inclusive $VH$ production cross section, its effect on the $M_{VH}$ distribution of the $ZH/WH$ ratio reaches the 20% level at $M_{VH}=600$ GeV, as illustrated in Fig. 6.^5^ In Ref. [7], an ${\mathcal O}(100\%)$ uncertainty on $\sigma _\gamma$ was estimated due to its strong dependence on the available PDF sets, implying a percent-level uncertainty on the total inclusive $VH$ production cross section. Due to recent theoretical progress in the determination of the photon PDFs [33], this source of uncertainty on $VH$ production has been significantly reduced to a level which allows us to neglect it in our analysis [34]. A variation of the electro-weak factorization scale by a factor of two around the central value of $M_\text {V}+M_\text {H}$ changes the electro-weak correction factor (including the photon-induced corrections) by less than 4% and would thus be invisible in Figs. 5 and 6.

**Fig. 7:**
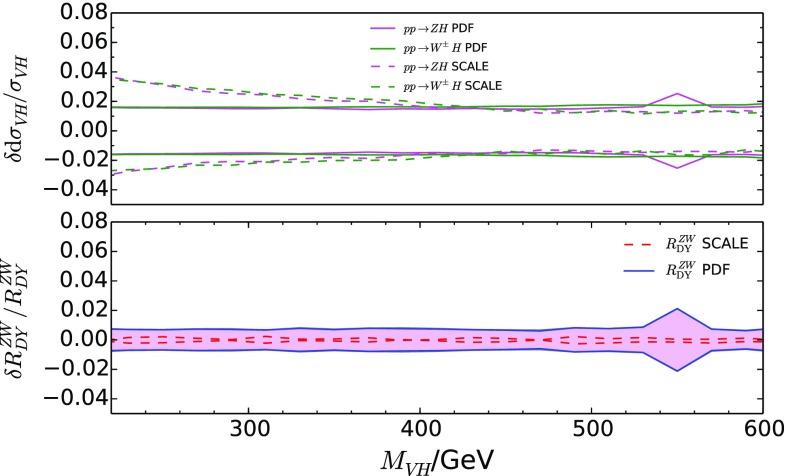
PDF uncertainty from the Monte-Carlo replicas (using the PDF4LHC15_nlo_mc PDF set with $\alpha _s(M_Z)=0.118$ [26]) and renormalization/factorization scale uncertainty ($\mu _\text {F}=\mu _\text {R}$ varied by a factor of two around $M_{VH}$), evaluated assuming full correlation between $WH$ and $ZH$, and using MC@NLO events with one emission added from the parton shower. This treatment is formally equivalent to an NLO calculation (see main text)

Let us next consider the uncertainties induced on $R_{\text {DY}}^{ZW}$ by the PDFs. While they amount to 2-4% on the cross sections themselves, they largely cancel in $R_{\text {DY}}^{ZW}$ when assuming that they are fully correlated between these two processes as demonstrated in Fig. 7. The uncertainties in this case have been calculated using MadGraph5_aMC@NLO [35, 36] MC@NLO events with one emission added through the HERWIG 7 parton shower [37, 38]. The single parton-shower emission re-introduces the NLO terms subtracted during the construction of the MC@NLO events, and hence this treatment is formally equivalent to an NLO calculation. The plot also includes the renormalization/factorization scale uncertainty, obtained by varying these scales by a factor of two around the central scale, where the latter is defined as half the sum of the transverse masses of all final state particles (including partons). We assume that these uncertainties are fully correlated between the $ZH$ and the $WH$ process, which is justified from the identical form of the DY-like QCD corrections for these two processess. The size of the scale variation on the ratio corroborates the observations from above about the uncertainties due to uncalculated higher-order QCD corrections. The PDF uncertainties for the set PDF4LHC15_nlo_mc [26] were calculated using the associated Monte-Carlo replicas. In our analysis below, we combine these uncertainties in quadrature.

### Estimate of the experimental uncertainty

In this section, we will provide a rough estimate of the uncertainty on the double ratio by combining the theoretical uncertainty on $R_{\text {DY}}^{ZW}$ with the experimental one on $R^{ZW}_{}$ through

9 $$\begin{aligned} \begin{aligned} \left( \frac{ \delta R_{R}^{ZW} }{ R_{R}^{ZW} } \right) ^2 = \left( \frac{ \delta R_{\text {DY}}^{ZW} }{ R_{\text {DY}}^{ZW} } \right) _\text {th}^2 + \left( \frac{ \delta R^{ZW}_{} }{ R^{ZW}_{} } \right) _\text {exp}^2 , \end{aligned} \end{aligned}$$

where the subscripts indicate that the first term is obtained through a theoretical calculation and the second through an experimental measurement. The quadratic sum of theoretical and experimental uncertainties is justified by the low level of correlation between the two. We assume total integrated luminosities for *pp* collisions at 13 TeV center-of-mass energy of (a) ${\mathcal L}=36.1~\hbox {fb}^{-1}$, (b) ${\mathcal L}=300~\hbox {fb}^{-1}$, and (c) ${\mathcal L}=3000~\hbox {fb}^{-1}$, corresponding to (a) the ATLAS luminosity underlying the analysis of Ref. [40], (b) the end of LHC Run 3, and (c) the future high-luminosity LHC run.

#### Details of the simulated analysis

We construct a hadron-level analysis, including decays of the vector bosons and the Higgs boson. The parton-level events for signal and backgrounds are generated at NLO using MadGraph5_aMC@NLO for all samples, except for $gg\rightarrow ZH$ which is generated at leading order. For all samples, we employed the PDF4LHC15_nlo_mc PDF set. Parton showering as well as hadronization and modeling of the underlying event is performed within the general-purpose Monte-Carlo event generator HERWIG 7. To take into account the higher-order corrections on $gg\rightarrow ZH$, we apply a global *K*-factor of $K=2$ [18, 20]. Electro-weak corrections largely cancel in the double ratio $R_R^{ZW}$ and can thus be neglected in our event simulation. We consider leptonic decays of the vector bosons, $W^\pm \rightarrow \ell ^\pm \nu _\ell$ and $Z\rightarrow \ell ^+ \ell ^-$, where $\ell = (e, \mu )$, and Higgs–Boson decays to $b{\bar{b}}$ pairs. As background processes we consider $pp \rightarrow t\bar{t}$, $pp \rightarrow W^\pm b \bar{b}$, $pp \rightarrow Z b \bar{b}$ and single top production. In this simplified phenomenological analysis, we do not consider any backgrounds coming from light jets which are mis-identified as *b*-jets, nor those coming from mis-identified leptons.^6^ To approximately take into account the NNLO corrections on the $pp \rightarrow t\bar{t}$ background, we apply a global *K*-factor of $K=1.2$ [41].

Jets are reconstructed using the anti-$k_T$ algorithm, implemented in the FastJet package [42, 43] with parameter $R=0.4$. The jet transverse momentum is required to be greater than 20 GeV for ‘central jets’ ($|\eta |<2.5$) and greater than 30 GeV for ‘forward jets’ ($2.5<|\eta |<5$). Selected central jets are labeled as ‘*b*-tagged’ if a *b*-hadron is found within the jet. A *b*-tagging efficiency of 70% is considered, flat over the transverse momentum of the jets, to reproduce the efficiency of the experimental *b*-tagging algorithm of Ref. [40]. The leading *b*-jet is required to have a transverse momentum larger than 45 GeV. The missing transverse energy is taken as the negative sum of transverse momenta of all visible particles. Electrons and muons are subject to isolation criteria by requiring the scalar sum of the transverse momenta of tracks in $R=0.2$ around them to be less than 1/10$^\text {th}$ of their transverse momentum: $\sum _{R< 0.2} p_{T}^{\mathrm {tracks}} < 0.1 \times p_{T}^\ell$.

#### Analysis strategy

The 13 TeV ATLAS analysis of Ref. [40] considered three event selections, corresponding to the $Z\rightarrow \nu \bar{\nu }$, the $W\rightarrow \ell \nu$, and the $Z\rightarrow \ell \ell$ channels. Here we only consider the latter two and refer to them as 1- and 2-lepton channel, respectively. All selections require exactly two *b*-tagged central jets, used to define the invariant mass $m_{b{\bar{b}}}$. For the $W\rightarrow \ell \nu$ selections, events with more than three central and forward jets are discarded.

In the $W \rightarrow \ell \nu$ analysis, the neutrino four-momentum is reconstructed by assuming that its transverse component is equal to the missing transverse momentum, $p^\nu _T = E_{T}^{\mathrm {miss}}$, and solving the quadratic equation $(p^\nu + p^\ell )^2 = M_\text {W}^2$ for the *z*-component $p^\nu _z$. The two resulting solutions can be used to construct two possible *W* four-momenta.^7^ These two *W* four-momenta are then combined with the *b*-jet candidates’ four-momentum, and the combination with invariant mass closest to the top mass is selected. This invariant mass, denoted by $m_\text {top}$, is used to suppress top quark-related backgrounds (see last cut below).

Further details on the 1-lepton and 2-lepton channels are as follows:

- $$Z\rightarrow \ell \ell $$ -channel:
  - exactly two same-flavor leptons (for muons: of opposite charge) with $p_{T}>7$ GeV and $|\eta |<2.5$, of which at least one has $p_{T}>25$ GeV;
  - lepton invariant mass 81 GeV $< m_{\ell \ell }< 101$ GeV;
  - $$p_{T}^{Z} > 150$$ GeV.
- $$W\rightarrow l\nu $$ -channel:
  - exactly 1 lepton with $p_{T}>25$ GeV and $|\eta |<2.5$;
  - $$p_{T}^{W} > 150$$ GeV;
  - $E_{T}^{\mathrm {miss}} > 30$ GeV in the electron sub-channel;
  - $m_{b{\bar{b}}} > 75$ GeV or $m_\text {top} \le 225$ GeV.

The events passing the selection cuts are subject to a “dijet-mass analysis”, following closely that of Ref. [40], where the BDT$_{VH}$ discriminant of the multivariate analysis is replaced by the invariant mass of the *b*-tagged jets, $m_{b{\bar{b}}}$. This results in ten signal regions, shown in the second and third rows of Table 12 in Ref. [40]. In the present analysis, we have only included signal regions with $p_{T}^{V} > 150$ GeV. We have further applied the requirement $m_{b{\bar{b}}} \in [110, 140]$ GeV which efficiently selects events containing $H\rightarrow b\bar{b}$. The expected number of events predicted by the Monte-Carlo level analysis at the selection level are similar to those of Ref. [40].

**Fig. 8:**
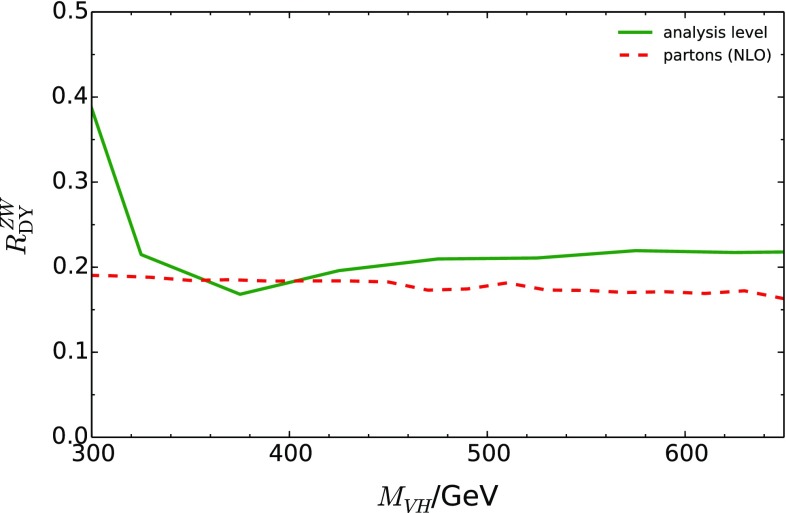
Comparison of the hadron-level prediction of the ratio of DY-like $ZH$-production to $WH$-production, $R_{\text {DY}}^{ZW}$, to the partonic prediction. (The curves in this plot include the branching ratios of the *Z* and the *W* boson.)

Figure 8 compares the hadron-level prediction, after analysis cuts, of the ratio $R_{\text {DY}}^{ZW}$ to the parton-level prediction. The parton-level prediction was constructed from the truth-level *W* and Higgs boson momenta, whereas the hadron-level curve was constructed through the combination of the reconstructed four-momenta of the *W* boson and the Higgs boson. For the *W* boson, a random choice was made between the two solutions for the *z*-component of the neutrino momentum. Figure 8 shows that this ratio is only moderately affected by the analysis and thus can be calculated fairly reliably within perturbation theory for the inclusive cross section. It is conceivable that the analysis could be modified appropriately to preserve more closely the parton-level form of $R_{\text {DY}}^{ZW}$.

**Table 1:** Numerical results for the double ratio $R^{ZW}_R$ and the associated statistical and systematic uncertainties, obtained by mimicking the analysis of Ref. [40]. The statistical uncertainty is evaluated for three values of the integrated luminosity ($\mathcal {L} = 36.1$, 300, and $3000\,\hbox {fb}^{-1}$). The systematic uncertainty is shown by assuming the individual systematic uncertainties of $ZH$ and $WH$ to be fully uncorrelated, moderately-correlated, and fully correlated, respectively ($p_{ZW} = (0, 0.5, 1.0)$). In the second line, the $VH$ invariant mass was restricted to $M_{VH}\in (350,650)$ GeV. The systematic uncertainties are assumed to be unchanged by this restriction

	$R_R^{ZW}$	Stat. (${\mathcal L}/\text {fb}^{-1}$)			Syst. ($p_{ZW}$)		
		36.1	300	3000	0	0.5	1
All $M_{VH}$	1.49	± 0.90	± 0.31	± 0.10	± 0.90	± 0.66	± 0.25
Restricted $M_{VH}$	1.55	± 1.08	± 0.38	± 0.12	± 0.90	± 0.66	± 0.25

## Numerical results

### Calculation of experimental uncertainties

The experimental ratio $R^{ZW}_{}$ is evaluated from

10 $$\begin{aligned} R^{ZW}_{}= \frac{{\mathrm {d}} N^{ZH}}{ {\mathrm {d}} N^{WH} } = \frac{ {\mathrm {d}} N^{\ell \ell } - {\mathrm {d}} N^{\ell \ell }_{\mathrm {bkg}} }{{\mathrm {d}} N^{\ell } - {\mathrm {d}} N^{\ell }_{\mathrm {bkg}}}, \end{aligned}$$

where ${\mathrm {d}} N^{X}$ and ${\mathrm {d}} N^{X}_{\mathrm {bkg}}$, with $X\in \{\ell ,\ell \ell \}$, represent the total number of events and the number of background events per bin, with $Xb{\bar{b}}$ final state, respectively. The uncertainty due to background subtraction will be included in the estimate of the overall systematic uncertainty. The uncertainty on $R^{ZW}_{}$ originating from the size of the total event samples expected to be collected is given by:

11 $$\begin{aligned} \left( \frac{ \delta R^{ZW}_{} }{ R^{ZW}_{} }\right) _{{\mathrm {stat.}}}^2= & {} \left( \frac{ \partial R^{ZW}_{} }{ \partial ({\mathrm {d}} N^{\ell \ell }) }\right) ^2 \delta ({\mathrm {d}} N^{\ell \ell })^2 \nonumber \\&+ \left( \frac{ \partial R^{ZW}_{} }{ \partial ({\mathrm {d}} N^{\ell }) } \right) ^2 \delta ({\mathrm {d}} N^{\ell })^2 . \end{aligned}$$

If we assume the expected number of events in each bin to be large enough, then ${\mathrm {d}} N^{X}$ is Gaussian-distributed with uncertainty $\delta ({\mathrm {d}} N^{X} ) = \sqrt{{\mathrm {d}} N^{X}}$, giving:

12 $$\begin{aligned} \left( \frac{ \delta R^{ZW}_{} }{ R^{ZW}_{} }\right) _{{\mathrm {stat.}}}^2 = \frac{ {\mathrm {d}}N^{\ell \ell } }{ ({\mathrm {d}} N^{\ell \ell } - {\mathrm {d}} N^{\ell \ell }_{\mathrm {bkg}})^2 } + \frac{ {\mathrm {d}} N^{\ell } }{ ({\mathrm {d}} N^{\ell } - {\mathrm {d}} N^{\ell }_{\mathrm {bkg}})^2 } \;. \end{aligned}$$

We define the systematic uncertainty on $R^{ZW}_{}$ to include all uncertainties which contribute to its experimental measurement. A precise determination of these systematics would require a full-fledged experimental analysis that would take into account all the correlations between the different contributing components. For the purpose of this paper, we content ourselves with an estimate of the uncertainty derived from the separate $ZH$ and $WH$ signal strengths of Eq. (13), presented in the ATLAS analysis of Ref. [40]:

13 $$\begin{aligned} \begin{aligned} \mu _{ZH}&= 1.12^{+0.34}_{-0.33}{\mathrm {(stat.)}}^{+0.37}_{-0.30}{\mathrm {(syst.)}},\\ \mu _{WH}&= 1.35^{+0.40}_{-0.38}{\mathrm {(stat.)}}^{+0.55}_{-0.45}{\mathrm {(syst.)}}. \end{aligned} \end{aligned}$$

The systematic uncertainty of these results includes all sources of experimental nature, related to the background and signal Monte-Carlo simulation and data driven estimates, and to the finite size of the simulated samples.

We assume that the (symmetrized) systematic uncertainties $(\delta \mu _{VH})_\text {syst.}$ can be propagated directly to the experimental ratio defined by Eq. (10), and thus to the double ratio:

14 $$\begin{aligned} \begin{aligned} \left( \frac{\delta R_R^{ZW}}{R_R^{ZW}}\right) ^2_\text {syst.} =\,&(\delta \mu _{ZH})_\text {syst.}^2 +(\delta \mu _{WH})_\text {syst.}^2\\&-2\,p_{ZW}\, (\delta \mu _{ZH})_\text {syst.}(\delta \mu _{WH})_\text {syst.}\\ =\,&0.112 + 0.250 - 0.335\,p_{ZW}. \end{aligned} \end{aligned}$$

where $p_{ZW}$ parameterizes the correlation of the systematic uncertainties between $ZH$ and $WH$ production. Table 1 shows the results for three different degrees of correlation: $p_{ZW} = 0$ (no correlation), $p_{ZW}=1/2$ (50% correlation), and $p_{ZW}= 1$ (full correlation).^8^

### Results semi-inclusive in $M_{VH}$

Let us first consider integrated quantities before turning to a more differential analysis below. From the hadron-level selection described in Sect. 3.3.1, it has been found that the analysis of Ref. [40] favors events with $M_{VH}\gtrsim 350$ GeV. Furthermore, we find that, in the present analysis, the $gg\rightarrow ZH$ process contributes substantially up to $M_{VH}\sim 650$ GeV. Therefore, we also present results where the events are restricted to $350~\text {GeV}< M_{VH}< 650~\text {GeV}$. Note that only the signal regions with $p_{T}^{V} > 150$ GeV are included.^9^

Due to the present rudimentary treatment of systematic uncertainties, these are only considered inclusively, and thus assumed unchanged by this restriction on the $M_{VH}$ range. Future experimental analyses, possessing information on the intricate correlations between systematics should be able to provide a more differential assessment. We present the results for the statistical and systematic uncertainties expected at integrated luminosities of ${\mathcal L}=36.1 / 300 / 3000~\hbox {fb}^{-1}$ in Table 1. From these numbers, one may evaluate the significance *s* to which the non-DY component can be observed through

15 $$\begin{aligned} \begin{aligned} s/\sigma&= \frac{\sigma ^{ZH}_\text {non-DY}}{\delta \sigma ^{ZH}_\text {non-DY}} = \frac{R_R^{ZW}-1}{\delta R_R^{ZW}}\\&= \frac{R_R^{ZW}-1}{\sqrt{(\delta R_R^{ZW})^2_\text {stat.} + (\delta R_R^{ZW})^2_\text {syst.}}}. \end{aligned} \end{aligned}$$

For $\mathcal {L}=3000\,\text {fb}^{-1}$, we thus find that the gluon-initiated component for $ZH$ production as predicted by the SM gives only a $2\sigma$ effect for the “restricted $M_{VH}$” sample assuming full correlation of the systematic errors between $ZH$ and $WH$ production. In case the systematic uncertainties can be decreased down to half the current value, the significance increases to $3.2\sigma$. Considering the fact that New-Physics models typically enhance the gluon-initiated component, a dedicated experimental analysis which is tailored to isolate this component and optimized for the $ZH/WH$ ratio measurement therefore seems appealing.

Let us take a moment to compare these results to the direct extraction of the non-DY component from $R_{\text {DY}}^{ZH}$ as sketched at the beginning of Sect. 3.1. In this case, we find a statistical error of $(\delta R_{\text {DY}}^{ZH})_\text {stat.} = 0.14\,(R_{\text {DY}}^{ZH}-1)$ in the restricted-$M_{VH}$ region, while the systematic error is given by $(\delta R^{ZH}_{\text {DY}})_\text {syst.}=R^{ZH}_{\text {DY}}(\delta \mu _{ZH})_\text {syst.}$ if we follow the analogous reasoning as above. Using our central value for the double ratio in the restricted-$M_{VH}$ region for $R_{\text {DY}}^{ZH}$, this leads to a signal significance of $1\sigma$. Assuming that the systematic uncertainty can be reduced by a factor of two, the significance for $R_{\text {DY}}^{ZH}\ne 1$ increases to $2\sigma$. Comparing this to $R_R^{ZW}$, we find that the direct measurement of $R_{\text {DY}}^{ZH}$ is competitive as long as the correlation between the systematic $ZH$ and $WH$ uncertainties is smaller than about 75%, i.e. roughly the value of $p_{ZW}$ where the correlation term in Eq. (14) cancels $(\delta \mu _{WH})_\text {syst.}$. At this point it is important to keep in mind that, as argued at the beginning of Sect. 3.1, we also expect significant contributions to the uncertainty from the theoretical input to $R_{\text {DY}}^{ZH}$, while they should be negligible for $R_R^{ZH}$. This means that already a significantly lower $ZH/WH$ correlation should lead to an improved extraction of the non-DY contribution by using the double ratio $R_R^{ZW}$.

### Results differential in $M_{VH}$

**Fig. 9:**
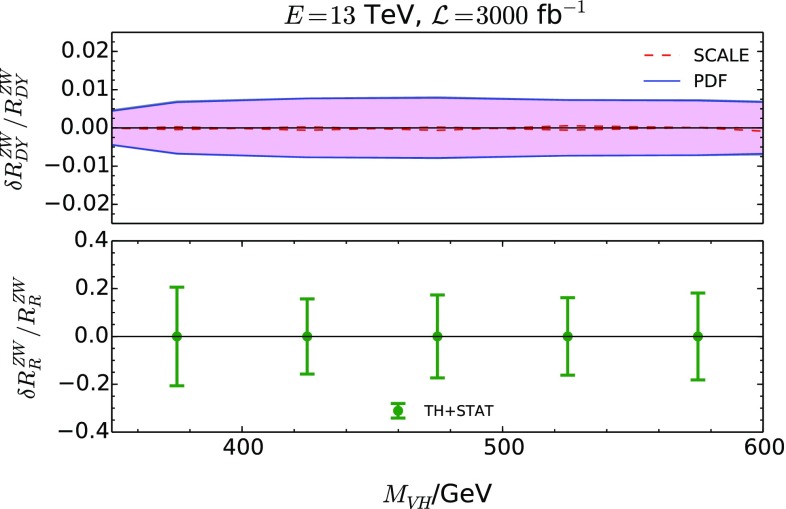
The upper panel shows the “theoretical” uncertainty, i.e. the first term in Eq. (9). In the lower panel, the green error bars show the total relative uncertainty as dictated by Eq. (9). The SM $gg\rightarrow ZH$ (with $K=2$) has been included in the “experimental” uncertainty. The invariant mass in the case of the $WH$ channel was constructed through the combination of the reconstructed four-momenta of the *W* boson and the Higgs boson. For the *W* boson, a random choice was made between the two solutions for the *z* component of the neutrino momentum

We now turn to the $M_{VH}$ distribution. Figure 9 shows the resulting fractional uncertainties coming from theory or data statistics as a function of the $VH$ system invariant mass. The upper panel shows the “theoretical” uncertainty, i.e. the first term in Eq. (9), obtained by considering the scale and PDF variations *after* applying the hadron-level analysis. In the lower panel, the error bars show the total uncertainty as dictated by Eq. (9), i.e. the combination of the theoretical and statistical uncertainties for an integrated luminosity of ${\mathcal L}=3000~\hbox {fb}^{-1}$, but excluding experimental systematic uncertainties. We refrain from assessing the latter as their differential behavior would be challenging to predict at this stage. It is evident that the statistical uncertainty originating from the equivalent data sample size for an integrated luminosity of ${\mathcal L}=3000~\hbox {fb}^{-1}$, ${\mathrm {d}} N^{X}$, dominates over the theoretical uncertainty.

**Fig. 10:**
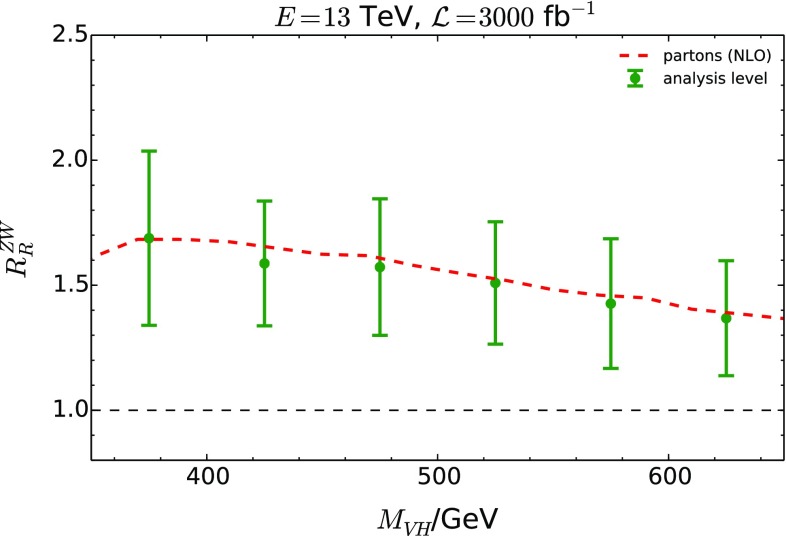
The double ratio $R_{R}^{ZW}$ is shown in green error bars, assuming SM $gg\rightarrow ZH$ (where we have applied a global *K*-factor of $K=2$). The size of error bars indicates the total theoretical and statistical uncertainty as given by Eq. (9). The red dashed line shows the inclusive double ratio at parton level, including the parton shower

Figure 10 demonstrates how an experimental measurement would look like, assuming that a $gg\rightarrow ZH$ component exists in the sample at the level of the central SM prediction.^10^ The double ratio $R_{R}^{ZW}$ is then given by:

16 $$\begin{aligned} R_{R}^{ZW} = 1 + \frac{ {\mathrm {d}} N^{ZH}_{gg} }{ {\mathrm {d}} N^{ZH}_{\text {DY}}}. \end{aligned}$$

Figure 10 also shows the theoretical parton-level distribution as red dashes (with no cuts applied). The theoretical prediction and experimental expectation are in good agreement in this range of the *VH* invariant mass. Note that the ATLAS analysis of Ref. [40] was not constructed to detect the $gg\rightarrow ZH$ component. It is thus conceivable that an analysis can be devised to increase its contribution to the total $ZH$ production with respect to the parton-level prediction.

## Conclusions

We have investigated New-Physics effects in the gluon-initiated Higgs-Strahlung process $gg\rightarrow ZH$ and have shown that the $ZH$ invariant mass distribution provides a particularly sensitive probe for physics beyond the SM. While the distribution below the $t\bar{t}$ threshold, $M_{ZH}< 2M_\text {t}$, remains rather unperturbed and thus may serve as a gauge for the experimental data, all New-Physics effects studied here can be clearly identified and to a large extent even distinguished by the kinematic region above that threshold. Recall that the low-$M_{ZH}$ region is also under fairly good theoretical control due to existing higher-order perturbative calculations in the large-$M_\text {t}$ limit [20]. Applying a phenomenological analysis at the hadronic level in order to estimate the expected theoretical uncertainty, we find that the SM $gg\rightarrow ZH$ component can be established at the $\sim 3.2\sigma$-level at the HL-LHC by comparing the experimental data to the theory prediction for the ratio of DY-like $ZH$ production to $WH$ production in the one- or two-lepton channels. Adding the zero-lepton channel and optimizing the current analyses for the $gg\rightarrow ZH$ process (or other non-DY processes) would most likely allow to reveal an ${\mathcal O}(5\sigma )$-level signal.

In order to uniquely establish New-Physics effects from this method, the theoretical control of the $gg\rightarrow ZH$ component needs to be further increased, for example by including SM top-mass effects at NLO. Considering the steady improvement of theoretical methods and existing calculations for very similar processes (see Ref. [45]), it is beyond doubt that this can be achieved in time for the analysis of HL-LHC data.

## Footnotes

1 Using PDF4LHC15_nnlo_100 [26].

2 Here and in what follows, we use the notation in Eq. (4) for a generic distribution. More explicitly, we may write  for a distribution in a specific kinematic variable $x\in \{p_{T}^H, M_{VH},\ldots \}$.

3 We note that, at the level of the actual Drell–Yan process of virtual *V* production, $pp\rightarrow V^*$, the symmetry between $V=W$ and $V=Z$ has been used before as an alternative way to measure the *W* boson mass at hadron colliders [31].

4 We use the default setting sbarelep=1 of HAWK for the final-state leptons, meaning that they are not re-combined with photons. For the *Z* decay into neutrinos, which is not considered in our analysis, the electro-weak corrections amount to about 10%.

5 Recall again that we only consider leptonic *Z* decays; for $Z\rightarrow \nu \bar{\nu }$, the effect of $\sigma _\gamma$ on the DY-ratio is even larger. Fig. 6 has been evaluated using the PDFs described in Ref. [33].

6 These are expected to be sub-dominant with respect to the ‘irreducible’ backgrounds, as is indeed the case in e.g. Ref. [40].

7 In the case of a negative discriminant in the quadratic equation, the $E_{T}^{\mathrm {miss}}$ vector is rescaled such that the discriminant becomes zero. The rescaling factor on the two $E_{T}^{\mathrm {miss}}$ vector components is chosen to be the same.

8 An earlier version of these results, based on lower statistics of our simulation, has been presented in Ref. [44].

9 Beyond enhancing the $gg\rightarrow ZH$ process contribution, this also ensures that the 1-lepton and 2-lepton analyses select similar phase space regions so as to facilitate cancellation of systematic uncertainties.

10 I.e., we use the SM $gg\rightarrow ZH$ cross section at $\mu _\text {F}=\mu _\text {R}=M_{VH}$, including a K-factor of $K=2$.
